# Shifting patterns in clinical pharmacy interventions associated with the integration of a clinical decision support system: A retrospective pre–post study in Brazil

**DOI:** 10.1371/journal.pone.0362555

**Published:** 2026-11-12

**Authors:** Tayná Letícia de Castro Santos, Patricia Carvalho Baruel Okumura, Christian Valle Morinaga, Pedro Kallas Curiati, Fábio de Cerqueira Lario

**Affiliations:** 1 Geriatric Emergency Department Research Group (ProAGE), Hospital Sírio-Libanês, São Paulo, São Paulo, Brazil; 2 Clinical Informatics Department, Hospital Sírio-Libanês, São Paulo, São Paulo, Brazil; Universita degli Studi di Siena, ITALY

## Abstract

**Introduction:**

Clinical decision support systems (CDSS) can enhance medication safety and optimize pharmacotherapy. This study aimed to evaluate the impact of implementing the Medi-Span CDSS, a rule-based artificial intelligence, on clinical pharmacy interventions in a tertiary care hospital.

**Methods:**

This retrospective analysis compared clinical pharmacy interventions before and after CDSS integration with a computerized physician order entry (CPOE) system using data from electronic health records. Exploratory analysis included the associations between CDSS alerts and clinical outcomes.

**Results:**

The proportion of hospitalizations with clinical pharmacy interventions increased after Medi-Span integration (13.4% vs. 11.2%, p < 0.01; adjusted OR=1.34, 95% CI: 1.26–1.41), with a shift toward therapeutic duplication (68.2% vs. 54.5%, p < 0.01) and a relative decrease in dose/posology interventions (44.6% vs. 54.8%, p < 0.01). Concurrently, unplanned intensive care unit admissions significantly decreased from 5.4% to 4.1% (p < 0.01), an association that remained significant in multivariate adjusted regression (OR=0.84, 95% CI: 0.77–0.91, p < 0.01; NNT = 118).

**Conclusion:**

The implementation of the Medi-Span CDSS in our CPOE was associated with more clinical pharmacy interventions, as well as a shift in their pattern.

## Introduction

Clinical pharmacists provide patient care to optimize pharmacotherapy, promote health and well-being, and prevent disease [1]. The goal is to increase the efficacy, safety, cost-effectiveness, and appropriate medication use [2]. According to Brazilian legislation, clinical pharmacists are responsible for participating in pharmacotherapy planning, evaluation and analysis of medication prescriptions [1]. Pharmacotherapy review can lead to interventions such as dose adjustments, suggestions for alternative pharmacotherapy, requests and monitoring laboratory tests related to pharmacotherapy, changes in schedules or drugs due to interactions and the discontinuation of therapeutic duplications [3,4].

Clinical pharmacists, similar to other health care professionals (HCP), can leverage information technology tools that have shown to prevent medication-related errors and reduce patient harm [5].The use of information systems, such as clinical decision support systems (CDSS) integrated to electronic health records (EHRs) and computerized physician order entry (CPOE), can assist pharmacists in making safer and more accurate decisions and ensure that patients receive the most appropriate treatment [6].

CDSS provide HCP with intelligently filtered information from EHRs presented at appropriate moments in the clinician workflow as suggestions to improve patient care [7,8]. They can generate alerts related to safety promotion, education, workflow improvement, effective communication, and quality of care [9,10]. Some CDSS provide clinicians with information without disrupting their workflow, such as prescription facilitators (suggestions for automatic patterns), access to relevant information (links to additional resources), and information displays (such as allergies or dosage alerts) [7,11]. Other tools have interruptive alerts, such as stop alerts and reminders requiring justification, which interrupt the workflow [7,12].

Pharmacists play a critical role in detecting and preventing medication-related errors, and the CDSS can enhance their proficiency in this area [2]. Many studies showing positive impact of a CDSS had a pharmacist as part of the multidisciplinary team, whereas unsuccessful interventions did not involve this professional [13]. In a systematic review published in 2018, Vélez-Diaz-Pallarés *et al*. concluded that the use of electronic prescriptions as a CDSS reduces medication-related errors, especially those related to dose, concentration, active ingredient, frequency of use, route of administration, and drug interactions [14]. The following year, in another systematic review, Roumeliotis *et al*. reported a reduction in medication-related errors and adverse events [15]. Recently, our group investigated the specific impact of Medi-Span alerts regarding potentially inappropriate medications in a targeted geriatric cohort, demonstrating an association with adverse events and prolonged length of stay [16]. However, whether the system-wide integration of such an active rule-based CDSS across a broader general adult inpatient population influences overall clinical pharmacy intervention rates, shifts intervention categories, or impacts hospital-wide patient outcomes remains unexplored.

Rule-based CDSS rely on software components, known as rule engines, that apply predefined conditional logic – such as “if/then” rules – to patient data [17]. By encapsulating expert medical knowledge into structured rule sets, these systems deliver transparent and interpretable alerts, diagnostic suggestions or treatment recommendations directly into the clinical workflow [18].

Medi-Span (Wolters Kluwer, Indianapolis, IN, USA) is a solution for drug data integration and automated clinical screening that provides continuously updated, evidence-based medication safety knowledge [19].. Medi-Span CDSS is a robust rule-based tool developed to support physicians and pharmacists in making better decisions related to medication prescription and dispensing, and alerts them about prescription errors, inappropriate doses, drug interactions and the risk of adverse events [19]. Medi-Span can actively integrate its knowledge engine into existing CPOE platforms – such as Philips Tasy® - to enable real-time alert generation during prescribing and dispensing workflows. Consequently, it has the strong potential to assist clinical pharmacists during their routine pharmacotherapy reviews by actively identifying dosing errors, interactions, therapeutic duplications, and opportunities to improve medication use, optimize treatment regimens, and ensure patient safety.

Although previous studies involving Medi-Span have evaluated its database for drug interactions [20], drug classification [21], pregnancy safety [22], off-label usage [23], medication pricing [24] and medication expenditure [25], most research predominantly addressed it merely as a passive drug information data source [20–25]. Indeed, its active, rule-based capabilities that alert HCP with treatment recommendations in real-time during the ordering workflow still lacks research, especially regarding application and impact in the hospital setting. Furthermore, there is a knowledge gap on how the active implementation of Medi-Span CDSS shapes clinical pharmacy interventions patterns and impacts major patient outcomes.

Therefore, this research aims to describe the implementation process of the Medi-Span CDSS into a major Brazilian hospital EHR, and evaluate its impact on clinical pharmacy interventions and patient outcomes before and after the integration.

## Methods

### Study design and setting

This study was a retrospective quasi-experimental, pre-post observational evaluation conducted at a philanthropic tertiary care hospital with 500 beds in São Paulo, Brazil. This institution serves a predominantly privately insured population and functions as a national center for highly complex cases.

### Study population and eligibility criteria

We analyzed all inpatient hospitalizations during two distinct 12-month observational periods:

- Pre-implementation period: August 1, 2019 to July 31, 2020.
- Post-implementation period: August 1, 2022 to July 31, 2023.

The two-year interval between August 2020 and July 2022 – representing phased CDSS deployment and the acute waves of the COVID-19 pandemic – was intentionally excluded to compare stable operational baselines.

Inclusion criteria: all hospitalizations of adult patients (≥18 years of age) admitted to critical care, emergency, or general inpatient units during the study timeframes. Exclusion criteria: pediatric hospitalizations (<18 years), outpatient visits. Regarding interventions, we included acceptable clinical pharmacy interventions and excluded those classified as administrative or logistical process interventions (unrelated to individual patient clinical parameters or drug safety rules, as these could not be influenced by the CDSS).

### Primary outcomes

The primary outcome was the occurrence and pattern of clinical pharmacy interventions. Interventions captured from the EHR were those accepted and classified into one of the three preventable categories potentially influenced by Medi-Span CDSS alerts:

- Dose/Posology: Adjustments to medication dose, administration frequency, or schedule, based on renal function, body weight, or age.
- Therapeutic Duplication: Identification of overlapping medications with the same therapeutic indication without synergistic intent, duplicate active ingredients across different routes, or failure to discontinue previous orders upon re-prescribing.
- Drug Interaction: Identification of clinically significant drug–drug or drug–food interactions (potential or manifested), or Y-site intravenous incompatibilities.

### Secondary outcomes

- Unplanned Intensive Care Unit (ICU) Admission: Binary outcome defined as an unexpected transfer from a general ward or emergency department to an ICU during the hospitalization.
- In-Hospital Mortality: Binary outcome defined as death occurring during the hospital stay

### Baseline covariates and data extraction

Demographic and clinical variables extracted included age (years), sex, total length of stay (days), admission via the Emergency Department (ED; binary), surgical procedure during hospitalization (binary), and comorbidity burden measured by the Charlson Comorbidity Index (CCI; continuous). Surgical status was extracted as a binary covariate indicating whether any surgical procedure under anesthesia was performed during the hospitalization.

Data were extracted automatically from secondary institutional databases between November 15 and December 15, 2023. Demographics, admission source, clinical outcomes, and CCI scores were obtained from the hospital Business Intelligence system. Clinical pharmacy interventions were extracted directly from the EHR pharmacy module (Tasy® - Philips). Medi-Span alerts were obtained from a database developed by the local information technology team. Principal investigators had access to patient identification. The analytic dataset was de-identified and anonymized prior to statistical modeling to ensure confidentiality of the individuals.

### Statistical analysis

Numerical variables were reported as mean and standard deviations. Categorical variables were reported in absolute counts and proportions. Normal distribution was verified through Kolmogorov-Smirnov test, means were compared through *t* test and proportions through X^2^.

Multivariate logistic regression was performed to explore the associations between Medi-Span use and the following binary clinical outcomes: [1] clinical pharmacy intervention [2], unplanned ICU admission, and [3] in-hospital mortality. Regression models were adjusted for possible confounding variables: age, comorbidity burden (as measured by the CCI), need for surgery, and prior emergency department admission. Adjusted Odds Ratio (aOR) with 95% confidence interval (95%CI) were reported.

To facilitate clinical interpretation of the multivariate logistic regression findings, the Number Needed to Expose (NNE) and Number Needed to Treat (NNT) were calculated for primary and secondary binary outcomes [26,27]. NNE/NNT reflects the average number of hospitalized patients exposed to the CDSS implementation required for one additional clinical pharmacy intervention to occur (or one unplanned ICU admission to be prevented) relative to the baseline pre-implementation period. All analyses were conducted in RStudio.

This study was approved by the Human Research Ethics Committee of Hospital Sírio-Libanês (CAAE 74249923.1.0000.5461) with a waiver of informed consent due to the retrospective analyses of anonymized secondary data.

## Results

### Medi-Span CDSS implementation process

Medi-Span CDSS was implemented in October 2020, into the existing CPOE system, developed locally and integrated with the EHR Tasy® - Philips. ICUs were the first to receive the tool. Implementation began with a two-week pilot in one critical unit, followed by two more critical units and one semi-critical unit for another two weeks. The alerts displayed by Medi-Span were classified as having high, moderate or low inconsistency, with high inconsistency being related to more critical information.

In the initially implemented workflow, high inconsistency alerts interrupted the workflows of both physicians (during the ordering process) and pharmacists (during review and order approval). To continue the workflow, HCP was required to justify it. In April, 2021 the project was interrupted for workflow adjustments, among them physician’s alerts became non interruptive with optional justification, whereas mandatory justification was retained for clinical pharmacists during order verification.

Integration process was resumed after adjustments in June 2021. Deployment was again gradual; first the system was tested in 50% of the hospital beds, and in August 2022, it was fully implemented. The system was integrated throughout the institution, covering critical, semi-critical, inpatient, and ED areas.

### Population demographics

Across the two study periods analyzed, a total of 6,144 unique patients accounted for 8,209 hospital admissions. During these admissions, clinical pharmacists performed a total of 19,118 interventions. Despite the two time periods having similar durations, the number of hospitalizations was higher during the post-Medi-Span period, with a statistically significant trend toward younger patients (58.9 versus 61.8 years, p < 0.01) with lower CCI values (2.1 versus 2.5, p < 0.01), higher rates of surgical interventions (55.8% versus 53.7%, p < 0.01), more frequently originating from the emergency department (40.5% versus 38.2%, p < 0.01), and fewer ICU admissions, both overall and unplanned (12.1% versus 14.8%, p < 0.01 and 4.1% versus 5.4%, p < 0.01, respectively). The magnitude of the difference between the groups was small but indicated that patients had less complexity in the post-implementation period (Table 1). Surprisingly, this period had a greater number of hospitalizations with clinical pharmacy interventions (13.4% versus 11.2%, p < 0.01).

**Table 1:** Total number and characteristics of hospitalizations in the two study periods.

	Pre-Medi-Span N = 19,297	Post-Medi-Span N = 29,699	P value
Hospitalizations with clinical pharmacy interventions, N (%)	2,165 (11.2)	3,979 (13.4)	<0.01
Sex (male), N (%)	10,077 (52.2)	15,362 (51.7)	0.22
Age (years), Mean (SD)	61.8 (19.6)	58.9 (19.1)	<0.01^a^
Length of stay (days), Mean (SD)	4.7 (14.0)	4.4 (19.5)	0.23 ^a^
Charlson Comorbidity Index^b^, Mean (SD)	2.5 (2.1)	2.1 (1.9)	< 0.01 ^a^
Surgery during hospitalization, N (%)	10,377 (53.7)	16,567 (55.8)	< 0.01
Admission from ED, N (%)	7,377 (38.2)	12,019 (40.5)	< 0.01
Death, N (%)	342 (17.7)	451 (15.2)	0.03
ICU admissions, N (%)	2,865 (14.8)	3,589 (12.1)	< 0.01
Non-planned ICU admissions, N (%)	1,053 (5.4)	1,218 (4.1)	< 0.01

SD = Standard Deviation; ED = Emergency Department; ICU = Intensive Care Unit; ^a^ through *t* test; ^b^ Range from 0 (none) to ≥5 (severe).

In multivariate analysis, the association between the use of Medi-Span and a greater number of clinical pharmacy interventions was shown to be statistically significant after controlling for eventual differences in clinical and demographic parameters between the groups (Table 2). The odds of a clinical pharmacy intervention were 34% higher after Medi-Span implementation, with a NNE of 31. That is, for every 31 new hospitalizations after this CDSS was implemented, there was one new additional clinical pharmacy intervention. A statistically significant reduction in unplanned ICU admissions was also demonstrated (OR = 0.84; 95% CI: 0.77–0.91; p < 0.01), with a NNT of 118.

**Table 2:** Multivariate analysis for the association of clinical outcomes and medi-span implementation.

	Odds Ratio*	95% Confidence interval	P value	NNE / NNT
Hospitalizations with clinical pharmacy interventions	1.34	1.26–1.41	<0.01	31
Non-planned ICU admissions	0.84	0.77–0.91	<0.01	118
Death	1.15	1.00–1.34	0.06	

ICU = Intensive Care Unit; NNE = Number Needed to Expose; NNT = Number Needed to Treat

* Adjusted for Age, Charlson Comorbidity Index, surgery during hospitalization and admission from ED

### Clinical pharmacy interventions

During the pre-implementation period, a total of 6,670 clinical pharmacy interventions were performed, whereas during the post-implementation period 12,448 interventions were performed. The categories that were considered preventable with the use of the tool in the way it was currently used were dose/posology, therapeutic duplication, and drug interaction (Table 3).

**Table 3:** Distribution of clinical pharmacy intervention categories by hospitalizations in the two study periods.

	Pre-Medi-Span N = 2,165	Post-Medi-Span N = 3,979	P value
Number of interventions per hospitalization, Mean (SD)	**2.15 (2.07)**	**2.42 (2.39)**	**< 0.01**
Dose/posology (N, %)	1,186 (54.8)	1,772 (44.6)	< 0.01
Therapeutic duplication (N, %)	**1,179 (54.5)**	**2,715 (68.2)**	**< 0.01**
Drug interaction (N, %)	9 (0.4)	86 (2.2)	0.07

During the study period, there was no significant increase in the number of clinical pharmacists (Fig 1), while there were some changes in the scope of work. Before the tool’s implementation, we had an average of 26 clinical pharmacists; in the post-intervention period, we had an average of 28.5 pharmacists. In 2022, the clinical pharmacy service schedule raised from 7am-7 pm to 7am-10 pm.

**Fig 1:**
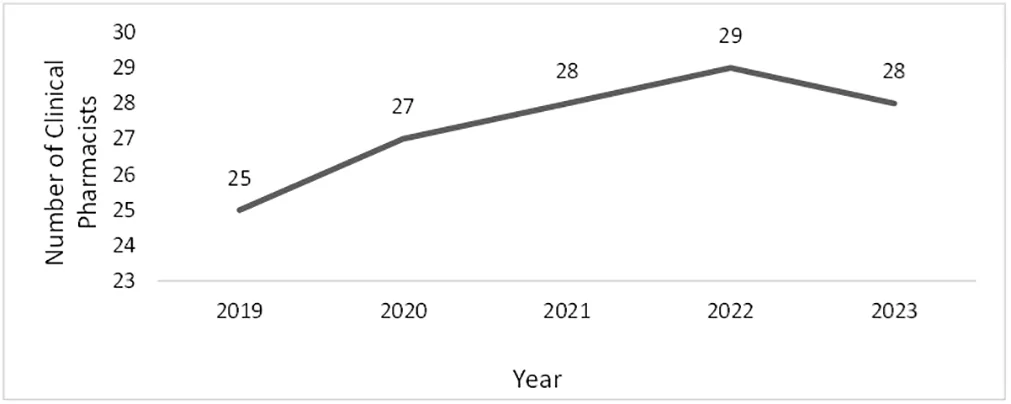
Number of Clinical Pharmacists in the Study Period.

## Discussion

This pre- and post-implementation study, involving approximately 50.000 hospitalizations across both periods, identified a shift on pharmaceutical practice that coincided with the integration of Medi-Span CDSS, regarding prescription adequacy and pharmacotherapy optimization. In particular, the results suggest that the use of Medi-Span CDSS is associated with reduction in complications that could lead to unplanned ICU admissions. This latest finding expands the knowledge about CDSS to improve drug prescription and minimize adverse events during hospitalization regarding previous studies.

A critical consideration in evaluating pre–post implementation studies is the potential confounding introduced by baseline shifts in patient case-mix. In our cohort, statistically significant demographic and clinical differences were observed between periods. However, contrary to the hypothesis that post-implementation increases in pharmacy interventions might be artificially driven by higher patient acuity, patients in the post-CDSS period were actually younger, had lower comorbidity burdens, and required fewer overall ICU admissions. Because lower clinical complexity typically reduces prescribing errors and intervention opportunities, this baseline imbalance acted as a conservative bias (biasing the effect size toward the null). The observed increase in intervention rates in a lower-acuity cohort — combined with a distinct qualitative shift toward therapeutic duplication alerts — suggests that the findings reflect genuine changes in workflow vigilance following CDSS integration rather than an artifact of patient severity.

The reduction in unplanned ICU admissions observed in the post-implementation period represents an encouraging clinical association. Clinically, while many minor drug-related problems (DRP) do not lead to severe harm, high-severity medications errors – such as unadjusted dosing of renally cleared toxic drugs, severe therapeutic duplications of central nervous systems depressants, or major anticoagulation interactions – can trigger catastrophic adverse drug events (e.g., acute kidney injury, severe hemorrhage, or acute respiratory failure). Preventing high-risk medication events through real-time CDSS alerts may contribute to averting acute clinical deterioration on general wards, directly associated with unplanned critical care transfer, prolonged length of stay and hospital-acquired infections [28]. Additionally, because the pre-implementation period only briefly overlapped with the early pandemic phase, it is unlikely that COVID-19 artificially inflated baseline unplanned ICU rates.

Our finding of increased pharmacy interventions post-CDSS resonates with the experience reported by Calloway *et al*. (2013) in a U.S. hospital. Their study documented a 105% increase in monthly clinical pharmacy interventions (from 1,986–4,065) following CDSS implementation [29]. Although Calloway *et al*. noted that other contemporaneous modifications within their pharmacy services could have influenced this outcome, they also highlighted an important associated benefit: the CDSS appeared to foster greater physician acceptance of the pharmacy team’s interventions. In the Brazilian context, Reis *et al*. (2013) previously demonstrated that routine clinical pharmacy reviews in a tertiary teaching hospital significantly intercept drug-related problems, predominantly involving dose adjustments and drug interactions [30]. However, while regional literature confirms the value of manual pharmaceutical reviews, studies evaluating active, real-time CDSS alerting tools integrated into hospital CPOE systems remain scarce in Latin America. Our results build upon these regional findings by demonstrating that implementing an active CDSS serves as a catalyst to expand the volume and efficiency of clinical pharmacy interventions within the Brazilian healthcare ecosystem.

The unexpected increase in clinical pharmacy interventions following Medi-Span implementation necessitates an examination of the tool’s integration within our institutional workflows and its interaction with physicians and pharmacists. Contrary to our initial hypothesis that a CDSS designed to support clinical decisions and reduce medication errors would decrease intervention rates, our findings highlight a more complex interplay. This counterintuitive finding may be contextualized by the literature, which emphasizes challenges such as alert fatigue and increased task completion times associated with CDSS implementation [31–33]. Notably, a systematic review by Hussain *et al*. (2019) revealed that CDSSs employing interruptive alerts for prescribers had the lowest acceptance rates [34].

A key factor potentially contributing to increased pharmacy interventions is the dichotomy in alert justification: optional for physicians versus mandatory for pharmacists regarding high-inconsistency alerts. This mandatory engagement likely heightened pharmacists’ scrutiny during prescription evaluation, thereby contributing to the greater number of documented interventions. Conversely, the optional nature of physician justification means that some alerts may have been overridden without detailed consideration, as previously reported [35]. However, data on physician alert acceptance rates or override rates were not collected, precluding an assessment of potential alert fatigue or underestimation of the tool by physicians.

While Medi-Span implementation was associated with a significant increase in clinical interventions for dose/posology and therapeutic duplication, the rise in interventions related to drug interactions did not reach statistical significance. This latter finding may reflect concerns highlighted in previous literature that CDSS-generated alerts for drug-drug interactions are often perceived as less clinically relevant, potentially leading to lower rates of pharmacist action on these specific alerts [36–38].

Finally, despite the overall increase in interventions per hospitalization, the distribution of these interventions shifted: there was a relative decrease in interventions for dose/posology and a substantial relative increase in interventions for therapeutic duplication. This shift is consistent with previous literature evaluating CDSS deployment. For instance, Rommers *et al*. (2011) demonstrated that implementing a computerized alerting system based on clinical rules shifted the pharmacist’s focus from conventional, time-consuming medication surveillance to more advanced therapeutic management, such as adjusting therapies based on complex patient parameters [39]. Rule-based advanced systems highlight complex clinical issues—such as drug-drug interactions, therapeutic duplications, and contraindications—thereby redirecting the clinical pharmacist’s expertise toward higher-level therapeutic optimization. We hypothesize that Medi-Span facilitates more proactive dose/posology adjustments by physicians, thereby reducing the proportion of such issues requiring pharmacist intervention, while simultaneously enhancing pharmacists’ detection of therapeutic duplications. Intriguingly, interventions for therapeutic duplication—triggered by ‘moderate’ (orange) alerts—increased proportionally more than those for dose/posology, which were associated with ‘high’ (red) alerts in our system (Fig 2). A more granular analysis, beyond the scope of this study, would be necessary to fully elucidate the reasons for this specific trend and the interplay between alert severity and pharmacist response.

**Fig 2:**
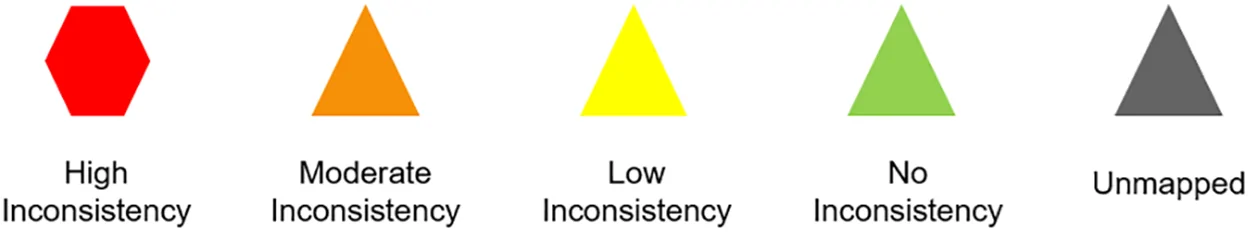
Medi-Span alerts classification.

This study has methodological limitations that require careful consideration. First, the use of secondary databases developed for purposes beyond this research, combined with the retrospective pre–post design is inherently vulnerable to temporal trends, unmeasured residual confounding, and differences in baseline patient case-mix. Furthermore, due to the secondary nature of the database, individual DRPs were not categorized using formal clinical severity scales. Additionally, surgical status was recorded as a binary indicator for risk adjustment, precluding a granular breakdown by surgical subspecialties or procedure acuity.

Although multivariate logistic regression was adjusted for key demographic and clinical covariates (age, comorbidity burden, surgical status, and emergency origin), statistical adjustment cannot fully rule out unmeasured confounders. Notably, patients in the post-implementation period had statistically lower clinical complexity (lower mean age, lower CCI, and lower ICU admission rates). Clinically, lower acuity would be expected to decrease the baseline indication for clinical pharmacy interventions; thus, the observed increase in interventions occurred despite a less complex patient population, suggesting that case-mix differences served as a conservative bias rather than an artificial driver of intervention rates.

Second, contemporaneous operational modifications occurred during the implementation timeframe, including an extension of clinical pharmacy coverage hours (from 12 to 15 hours daily) and a modest increase in average staffing (from 26 to 28.5 pharmacists). These structural improvements likely facilitated increased intervention documentation independently of or synergistically with CDSS integration. Third, due to the secondary nature of the database, data on physician alert acceptance or override rates were unavailable, precluding direct measurement of prescriber alert fatigue. Consequently, our findings represent real-world observational associations within a dynamic clinical environment rather than definitive causal effects of CDSS deployment.

## Conclusion

The implementation of the Medi-Span CDSS was associated with an unexpected increase in clinical pharmacy interventions – characterized by a notable shift toward therapeutic duplication — and coincided with a reduction in unplanned ICU admissions. Rather than establishing direct causality, these findings illustrate how automated alert tools coexist with evolving multidisciplinary workflows and structural service changes in a tertiary hospital setting.

Based on the limitations identified in this study, some recommendations emerge for future research. First, future studies should consider prospective designs, such as cluster-randomized controlled trials or stepped-wedge implementations across different hospital units. Second, employing advanced quasi-experimental analytical methods, such as propensity score matching, could more robustly control for residual confounding and baseline case-mix differences. Finally, future multi-center research should evaluate the long-term impact of active CDSS deployment on hard clinical outcomes — including drug-related adverse events, hospital length of stay, and readmission rates — while directly measuring physician alert acceptance and override behaviors to further elucidate the causal pathways between CDSS alerts and clinical outcomes.
